# Investigating the Mechanism of Emodin in Rheumatoid Arthritis Through the HIF-1α/NLRP3 Pathway and Mitochondrial Autophagy

**DOI:** 10.3390/cimb47070486

**Published:** 2025-06-25

**Authors:** Dehao Du, Linlan Zhou, Jiayu Tian, Lianying Cheng, Han Zhang, Yifu Tang, Zexuan Qiu, Tingdan Zhang, Xiaofeng Rong

**Affiliations:** 1 Department of Integrated Traditional Chinese and Western Medicine, Sci-Tech Innovation Center, The First Affiliated Hospital of Chongqing Medical University, Chongqing 400016, China; dudehao715@163.com (D.D.); zhoulinlan1235pm@163.com (L.Z.); jiayutian0116@163.com (J.T.); chenglianying2022@163.com (L.C.); 13067038559@163.com (Z.Q.); 15223413265@163.com (T.Z.); 2 School of Basic Medical Sciences, Chongqing Medical University, Chongqing 400016, China; zhanghanxz21@163.com (H.Z.); w4wy4y@163.com (Y.T.)

**Keywords:** rheumatoid arthritis, emodin, cobalt chloride, hypoxia, pyroptosis, mitochondrial autophagy

## Abstract

In this study, we investigated the inhibitory effects of emodin on pyroptosis in rheumatoid arthritis (RA) synovial cells by modulating the HIF-1α/NLRP3 inflammasome pathway and mitochondrial autophagy. By employing a chemically induced hypoxia model with CoCl_2_, we established experimental groups including normal control, model group, and emodin-treated groups at different concentrations (5 μM, 10 μM, and 20 μM). We optimized the CoCl_2_ concentration via CCK-8 assay to ensure cell viability. ELISA, Western blotting, transmission electron microscopy, and immunofluorescence were employed to assess HIF-1α, IL-1β, and IL-18 levels, pyroptosis-related proteins, autophagy markers, and NLRP3 fluorescence intensity. Statistical analysis revealed that increased CoCl_2_ concentrations led to a significant cell viability reduction (*p* < 0.05), with 300 μM CoCl_2_ causing ~50% inhibition at 24 h. Transmission electron microscopy confirmed autophagosome formation in emodin-treated groups, while Western blotting showed dose-dependent downregulation of HIF-1α, NLRP3, BNIP3, and related proteins. Immunofluorescence revealed reduced NLRP3 fluorescence intensity with increasing emodin doses (*p* < 0.05), alongside dose-dependent cell viability recovery (*p* < 0.05). Our findings demonstrate that emodin alleviates RA synovitis through dual mechanisms: inhibition of mitochondrial autophagy to regulate the balance between mitochondrial autophagy and pyroptosis, and suppression of HIF-1α/NLRP3-mediated pyroptosis signaling, thereby reducing IL-1β and IL-18 release and inhibiting synovial cell proliferation. This study provides innovative approaches for targeted RA therapy.

## 1. Introduction

Rheumatoid arthritis (RA) is a systemic autoimmune disease characterized by chronic synovitis and progressive joint destruction. Its pathogenesis involves the interplay of genetic susceptibility, age-related immune dysregulation, and environmental factors [1,2]. Current research primarily focuses on bone erosion mechanisms directly mediated by inflammatory responses, while pathological processes triggered by hypoxia in the synovial microenvironment, such as pyroptosis, have not been adequately explored.

The pathological features of RA include synovitis, with a key pathogenic mechanism being a significant decrease in oxygen partial pressure in synovial tissues. This decrease is primarily due to the abnormal proliferation of synoviocytes and increased metabolic oxygen consumption caused by inflammatory cell infiltration [3,4]. In this hypoxic microenvironment, hypoxia-inducible factor-1α (HIF-1α) abnormally accumulates, forming a pathological signaling hub [5]. Recent studies have shown that HIF-1α plays a critical role in RA progression by dynamically regulating the balance between pyroptosis and mitophagy (mitochondrial autophagy) [6].

For CoCl_2_, a commonly used hypoxia inducer, it enhances the expression of HIF-1α in a dose- and time-dependent manner in vitro [7], mirroring results observed under hypoxic conditions. Its mechanism involves Co^2+^ activation of two signaling pathways: PI3K/Akt/mTORC1 and MEK/ERK, which stimulate the production of reactive oxygen species (ROS), a crucial factor for HIF-1α activation [8,9]. Moreover, PI3K/Akt/mTORC1 facilitates the translation of HIF-1α mRNA. PI3K activates Akt, which subsequently phosphorylates mTORC1 in mammals, thereby activating downstream effectors that bind to the transcription initiation site of HIF-1α mRNA, enhancing protein translation [8]. Additionally, during the oxygen-dependent hydroxylation reaction of prolyl hydroxylase domain (PHD) proteins on hypoxia-inducible factor (HIF)-α subunits, Co^2+^-induced ascorbate depletion favors the redox state of intracellular Fe^3+^, resulting in the inactivation of PHD activity and stabilization of HIF-1α [10]. HIF-1α induces pyroptosis via the HIF-1α/NLRP3 pathway. Pyroptosis is a form of programmed cell death initiated by inflammasomes, characterized by the progressive swelling of cells until the cell membrane ruptures. This rupture releases inflammatory factors that trigger or exacerbate inflammatory responses [11].

In our previous study, we demonstrated that emodin, an active component of rhubarb (a traditional Chinese medicine), can effectively slow down the progressive destruction of joint structures by inhibiting the activation of the MAPK and NF-κB signaling pathways in synovial fibroblasts and modulating the RANKL/OPG axis balance [12,13,14,15]. This study aims to further investigate whether emodin exerts its effects by regulating the HIF-1α/NLRP3 inflammasome pathway and the mitophagy process, thereby inhibiting the expression of pyroptosis-related cytokines such as IL-1β and IL-18. The goal is to provide experimental evidence for elucidating the multi-target anti-RA effects of this natural compound.

## 2. Materials and Methods

### 2.1. Experimental Materials

#### 2.1.1. Experimental Cells

Human RA synovial fibroblasts (MH7A cells) were selected for this experiment, purchased from Biovector NTCC company (Beijing, China).

#### 2.1.2. Experimental Reagents

Emodin was purchased from MedChemExpress (Monmouth Junction, NJ, USA); CoCl_2_ was purchased from SIGMA (Darmstadt, Germany); HIF-1α, IL-1β, and IL-18 ELISA kits were purchased from Jiubang Bio (Quanzhou, China); HIF-1α, NLRP3, ASC, GSDMD, BNIP3, and TOM20 were purchased from Wuhan Sanying (20960-1-AP, 30109-1-AP, 10500-1-AP, 20770-1-AP, 68091-1-Ig, 11802-1-AP) (Wuhan, China); LC3B and Caspase-1 were purchased from Abmart (T55992, T510200) (Shanghai, China).

### 2.2. Experimental Methods

#### 2.2.1. Cell Culture

MH7A cells were routinely cultured in DMEM high-glucose medium containing 100 U/mL penicillin, streptomycin, and 10% fetal bovine serum in an incubator with 5% CO_2_ at 37 °C, and constant temperature and humidity.

#### 2.2.2. CCK-8 Assay to Detect Cell Viability

CoCl_2_ was used to establish a hypoxia model of MH7A cells [7]. Cells were cultured in a 96-well plate at a density of 4000 cells per well, followed by the addition of complete medium containing CoCl_2_ at different gradient concentrations (100 μM, 200 μM, 300 μM, 400 μM, and 500 μM). Cells were incubated in a constant temperature incubator at 37 °C for 12 h, 24 h, and 36 h, respectively. After stimulation treatment, diluted CCK-8 reagent was added and incubated with cells for 1 h. Subsequently, absorbance values were measured at a wavelength of 450 nm using a microplate reader, and cell activity was expressed as cell viability calculated by the following formula:

Cell viability = [(OD_exp_ − OD_st_)/(OD_nrm_ − OD_st_)] × 100%

where OD_exp_ = absorbance of the experimental group, OD_nrm_ = absorbance of the normal group, OD_st_ = absorbance of the blank group.

The CCK-8 assay was employed to evaluate the viability of cells treated with emodin (EMO). Cells were exposed to emodin at concentrations of 5, 10, 20, 30, 40, and 50 μM. In a 96-well plate, cells were cultured at a density of 4000 cells per well. Once the cells had adhered, emodin was added at the specified concentrations. The cells were incubated at 37 °C in a constant temperature incubator for 24 h. Subsequently, the diluted CCK-8 reagent was introduced and allowed to incubate with the cells for 1 h. Finally, absorbance was measured at 450 nm using a microplate reader, and cell viability was calculated.

CCK-8 assay was used to measure cell activity after drug administration, dividing cells into a normal group, model group, low-dose emodin group (5 μM), medium-dose emodin group (10 μM), and high-dose emodin group (20 μM). Cells were cultured in a 96-well plate at a density of 4000 cells per well, followed by the addition of 300 μM CoCl_2_ and corresponding concentrations of emodin (5 μM, 10 μM, and 20 μM). Cells were incubated in a constant-temperature incubator at 37 °C for 24 h. Afterward, the diluted CCK-8 reagent was added and incubated with cells for 1 h. Finally, absorbance was measured at 450 nm using a microplate reader, and cell viability was calculated. The dosage gradient was selected based on the on the non-cytotoxic concentration range reported in the literature [16], choosing 5–20 μM for this experiment.

#### 2.2.3. ELISA Assay for Detecting HIF-1α, IL-1β, IL-18 Levels in Cells

The culture supernatant of each group of cells was collected and centrifuged at 2500 rpm for 20 min at 4 °C. After centrifugation, the supernatant was collected again, and the levels of HIF-1α, IL-1β, and IL-18 were detected using ELISA kits.

#### 2.2.4. Scanning Electron Microscopy to Observe Cell Pyroptosis

A scanning electron microscope (SEM) was used to observe the morphology of normal cells and cells subjected to hypoxia treatment induced by CoCl_2_. SEM-specific cover slips were placed in a six-well plate with 1.5 × 10^5^ cells seeded per well, and cultured in a 37 °C, 5% CO_2_ incubator for 24 h to allow cells to adhere and fully extend. The experimental groups were set as normal and model groups. Both groups were treated for 24 h under the same culture conditions. After the cells formed a monolayer, they were rinsed three times with PBS, each time for 3 min. Subsequently, PBS was aspirated dry, and fixative was gently added along the well wall. After PBS washing, cells were fixed with 2.5% glutaraldehyde in 0.1 M phosphate buffer (pH 7.4) for 2 h at 4 °C. Samples were then dehydrated through an ethanol gradient (30%, 50%, 70%, 90%, and 100%, × 3), critical-point dried with liquid CO_2_, and sputter-coated with 10 nm gold–palladium. Finally, the samples were sent to the Electron Microscopy Center at Chongqing Medical University for observation.

#### 2.2.5. Transmission Electron Microscopy for Detecting Cell Autophagosomes

Cell samples were collected from five groups: normal, model, low-dose emodin (5 μM), medium-dose emodin (10 μM), and high-dose emodin (20 μM) using the scraping method. The samples were centrifuged at 4 °C at 1200 rpm for 10 min, then the supernatant was removed and the cells were fixed in glutaraldehyde at 4 °C overnight. The cells were post-fixed with 1% osmium tetroxide for 1 h and the entire sample was stained with 2% uranyl acetate. After dehydration through a graded ethanol series, the samples were embedded in EPON 812 resin. A Leica UC7 ultramicrotome (Leica Microsystems, Wetzlar, Germany) was used to cut ultrathin sections of 70 nanometers, which were stained with lead citrate and then observed at the Electron Microscopy Center of Chongqing Medical University.

#### 2.2.6. Western Blot Method to Detect Protein Expression of HIF-1α, NLRP3, BNIP3 in MH7A Cells

Cells treated in each group were collected, and protein concentration was measured using the BCA method. Appropriate amounts of dilution and loading buffer were added to samples and boiled at 95 °C for 10 min. A quantity of 10 μL of the sample was loaded for electrophoresis, transfer, and blocking steps. After washing the membrane with TBST buffer, primary antibody dilution was added, diluting HIF-1α, ASC, Caspase-1, GSDMD, NLRP3, BNIP3, TOM20, and LC3B (dilution ratio 1:1000), and incubated overnight at 4 °C. Subsequently, the membrane was washed with TBST, secondary antibody was added for incubation, and the membrane was washed again with TBST. ECL working solution was added, and exposure imaging was performed under a fully automatic imaging analyzer. Finally, results were statistically analyzed using ImageJ software (version 15.4F) provided by the National Institutes of Health, Bethesda, MD, USA.

#### 2.2.7. Immunofluorescence Staining Method to Detect NLRP3 Expression

MH7A cells were cultured to approximately 70–80% confluence, and the old medium was discarded, and replaced with medium containing different concentrations of emodin and CoCl_2_. The model group was replaced with medium containing only CoCl_2_, and co-cultured for another 24 h. After 24 h, the supernatant was discarded, and cells were washed with PBS and fixed with 4% paraformaldehyde for 1 h. After fixation, the cells were washed and treated with 0.5% Triton X-100 for 10 min. Then, they were blocked with a serum-containing blocking solution at room temperature for 30 min. Subsequently, primary antibody was added and incubated overnight at 4 °C. The next day, the cells were washed, and fluorescently labeled secondary antibody was added; the cells were then incubated at 37 °C for 1 h, and washed with PBST. Finally, nuclei were counterstained with DAPI for 5 min, and fluorescence signals were collected using a laser confocal microscope.

#### 2.2.8. Statistical Analysis

Statistical assessment and data visualization were performed using GraphPad Prism version 10.0 software (GraphPad Software, San Diego, CA, USA). Each experiment was conducted in triplicate. One-way analysis of variance (ANOVA) was used to evaluate statistical significance, followed by Dunnett’s test to compare with the model control group (MC). Tukey’s test was applied for comparisons between different doses within the emodin treatment groups. Quantitative results are expressed as mean ± standard deviation (SD), with a *p*-value of less than 0.05 considered statistically significant.

## 3. Results

### 3.1. MH7A Cell Viability After CoCl_2_ Induction

**Figure 1:**
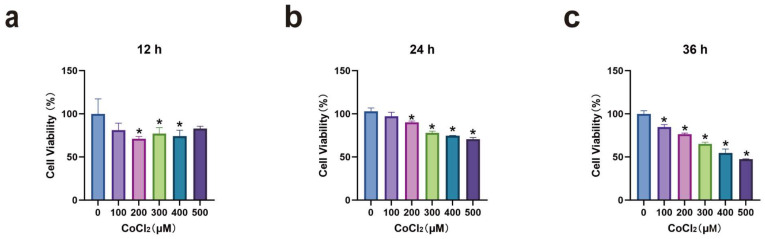
Effect of CoCl_2_ on cell viability detected by CCK-8 method: (**a**): Cell viability after 12 h induction: 200, 300, 400 μM CoCl_2_ groups, * *p* < 0.05; (**b**): cell viability after 24 h induction: 200, 300, 400, 500 μM CoCl_2_ groups, * *p* < 0.05; (**c**): cell viability after 36 h induction: 100, 200, 300, 400, 500 μM CoCl_2_ groups, * *p* < 0.05.

The effect of CoCl_2_ on MH7A cell viability was assessed using CCK-8 assay. Results indicated that as CoCl_2_ concentration and treatment duration increased, MH7A cell viability generally decreased. A minimal impact on cell activity was observed at 12 h, whereas excessive inhibition occurred at 36 h. The inhibition rate of cell activity with 300 μM CoCl_2_ at 24 h was approximately 75%. Consequently, subsequent experiments utilized 300 μM CoCl_2_ for 24 h treatments (*p* < 0.05) (see Figure 1).

### 3.2. Morphological Changes of MH7A Cells After CoCl_2_ Induction

**Figure 2:**
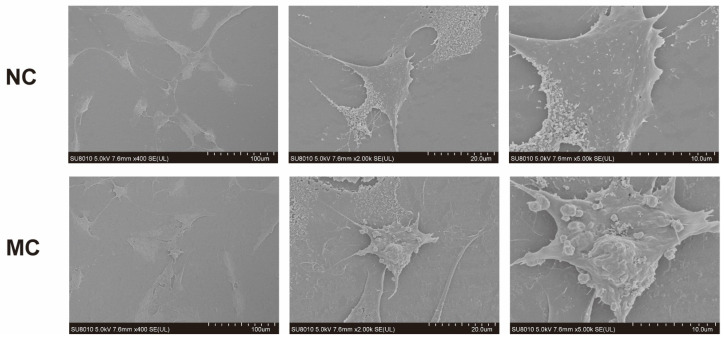
Scanning electron microscopy observation of cell pyroptosis. Cells from the normal group and model group were observed and photographed at 400×, 2000×, and 5000× magnifications under an electron microscope.

Under scanning electron microscopy, normal MH7A cells exhibited a triangular or irregular shape with rough edges. Following hypoxia induction with CoCl_2_, cell pyroptosis was observed, characterized by cell swelling and numerous bubble-like protrusions (see Figure 2).

### 3.3. CCK-8 Detection of Cell Viability in Each Group

CCK-8 assay results showed that high doses of emodin (30, 40, and 50 μM) inhibited cell viability by nearly 50% (*p* < 0.05), whereas low doses of emodin also inhibited cell viability but maintained viability above 75%.

**Figure 3:**
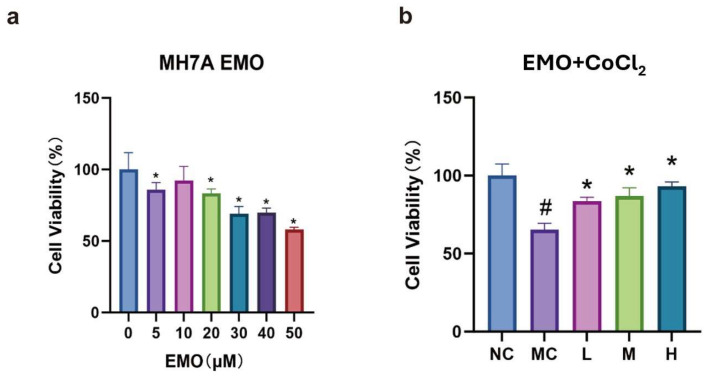
(**a**) Cell viability affected by emodin alone; (**b**) cell viability under the combined effect of emodin and CoCl_2_. NC: normal group, MC: model group (CoCl_2_: 300 μM), L: low-dose emodin group (CoCl_2_: 300 μM, Emo: 5 μM), M: medium-dose emodin group (CoCl_2_: 300 μM, Emo: 10 μM), H: high-dose emodin group (CoCl_2_: 300 μM, Emo: 20 μM). In graph a, compared to the normal group, # *p* < 0.05 for the model group; compared to the model group, * *p* < 0.05 for the emodin groups.

The CCK-8 assay results indicated that, compared to the normal group, cell viability in the model group significantly decreased (*p* < 0.05). In contrast, cell viability in each emodin dosage group significantly increased compared to the model group (*p* < 0.05). The high-dose emodin group exhibited significantly higher cell viability than the low-dose group (*p* < 0.05), while there was no significant difference between the medium and low doses or between the medium and high doses (*p* > 0.05; see Figure 3).

### 3.4. Expression Levels of HIF-1α, IL-1β, and IL-18 in Each Group of Cells

**Figure 4:**
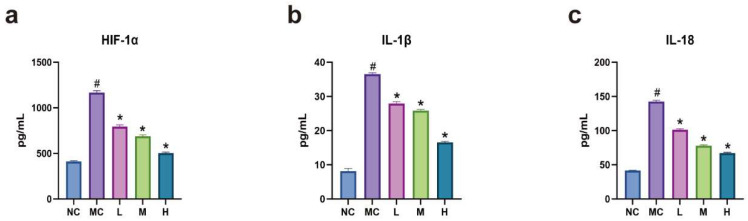
(**a**–**c**) ELISA results for detecting HIF-1α, IL-1β, and IL-18: NC: normal group, MC: model group (CoCl_2_: 300 μM), L: low-dose emodin group (CoCl_2_: 300 μM, Emo: 5 μM), M: medium-dose emodin group (CoCl_2_: 300 μM, Emo: 10 μM), H: high-dose emodin group (CoCl_2_: 300 μM, Emo: 20 μM). Compared to the normal group, # *p* < 0.05 for the model group; compared to the model group, * *p* < 0.05 for the emodin groups.

ELISA results revealed that, compared to the normal group, levels of HIF-1α, IL-1β, and IL-18 were significantly elevated in the model group (*p* < 0.05). In contrast, these levels were significantly reduced in each emodin dosage group compared to the model group (*p* < 0.05). Furthermore, as the emodin dosage increased, the reduction in the expression of HIF-1α, IL-1β, and IL-18 became more pronounced (*p* < 0.05). This indicates that emodin can inhibit the release of inflammatory factors such as HIF-1α, IL-1β, and IL-18 (see Figure 4).

### 3.5. Observation of Mitochondrial Autophagy in MH7A Cells Using Electron Microscopy

**Figure 5:**
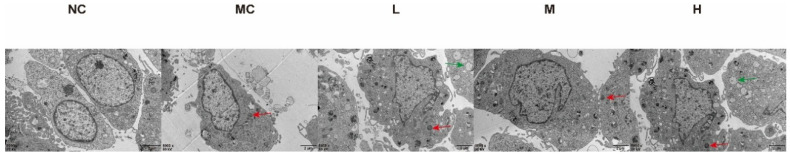
Electron microscopy observation of mitophagy in MH7A Cells: NC: normal group, MC: model group (CoCl_2_: 300 μM), L: low-dose emodin group (CoCl_2_: 300 μM, Emo: 5 μM), M: medium-dose emodin group (CoCl_2_: 300 μM, Emo: 10 μM), H: high-dose emodin group (CoCl_2_: 300 μM, Emo: 20 μM).

Transmission electron microscopy was utilized to observe mitochondrial autophagy. Qualitative analysis revealed mitochondrial autophagy in the model group cells, with autophagosomes (indicated by red arrows) present in each emodin dosage group. Endoplasmic reticulum expansion due to hypoxia was observed in both the low- and high-dose emodin groups (indicated by green arrows; see Figure 5).

### 3.6. Expression of Pyroptosis-Related Proteins in Each Group of Cells

**Figure 6:**
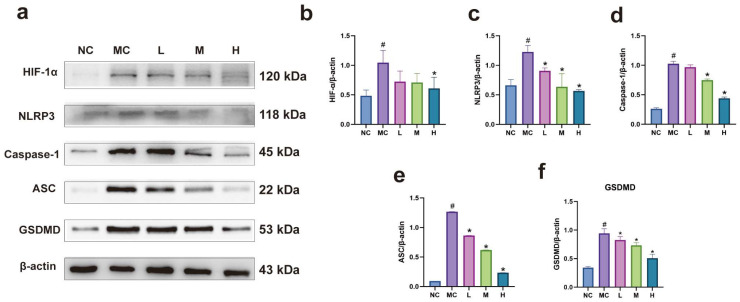
(**a**) Western blot band image; (**b**–**f**) bar charts illustrating statistical data on the expression of pyroptosis-related proteins: NC: normal group, MC: model group (CoCl_2_: 300 μM), L: low-dose emodin group (CoCl_2_: 300 μM, Emo: 5 μM), M: medium-dose emodin group (CoCl_2_: 300 μM, Emo: 10 μM), H: high-dose emodin group (CoCl_2_: 300 μM, Emo: 20 μM). Compared to the normal group, # *p* < 0.05 for the model group; compared to the model group, * *p* < 0.05 for the emodin groups.

Western blot results indicated that, compared to the normal group, the expression of proteins such as HIF-1α, NLRP3, and GSDMD was significantly increased in the model group (*p* < 0.05). In contrast, expression levels of these proteins were significantly decreased in each emodin dosage group compared to the model group (*p* < 0.05). There was no significant difference in HIF-1α expression among the emodin dosage groups (*p* > 0.05). Compared to the low-dose group, NLRP3 and GSDMD levels were significantly reduced in the high-dose emodin group (*p* < 0.05), with no significant differences observed between the medium and low-dose groups or the medium- and high-dose groups (*p* > 0.05). Significant differences were found in Caspase-1 and ASC levels among the emodin dosage groups, with the most pronounced decrease occurring in the high-dose group (*p* < 0.05). These findings suggest that emodin can inhibit cell pyroptosis mediated by the HIF-1/NLRP3 pathway (see Figure 6).

### 3.7. Expression of NLRP3 in Each Group of Cells

**Figure 7:**
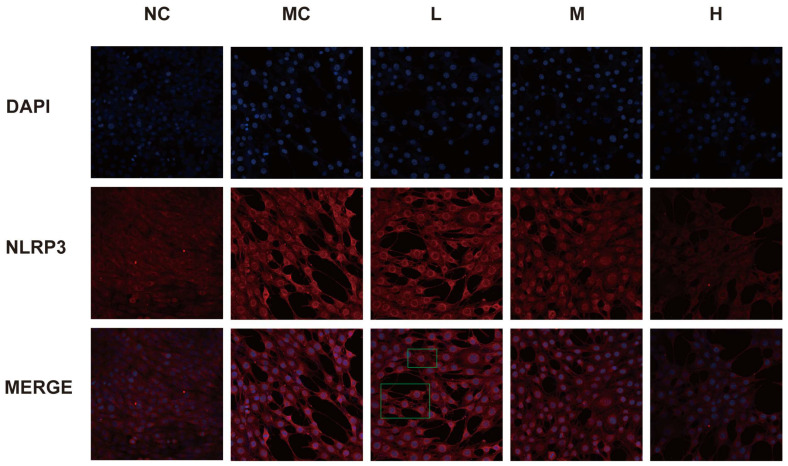
NLRP3 fluorescence results: NC: normal group, MC: model group (CoCl_2_: 300 μM), L: low-dose emodin group (CoCl_2_: 300 μM, Emo: 5 μM), M: medium-dose emodin group (CoCl_2_: 300 μM, Emo: 10 μM), H: high-dose emodin group (CoCl_2_: 300 μM, Emo: 20 μM).

Immunofluorescence results indicated that, compared to the normal group, fluorescence intensity in the model group was significantly increased. In contrast, fluorescence intensity in each emodin dosage group was markedly reduced. As the concentration of emodin increased, the fluorescence intensity of NLRP3 gradually decreased (*p* < 0.05). Pyroptotic cells were visible in the low-dose emodin group (highlighted within the green box; see Figure 7).

### 3.8. Expression of BNIP3 and LC3B Proteins in Each Group of Cells

**Figure 8:**
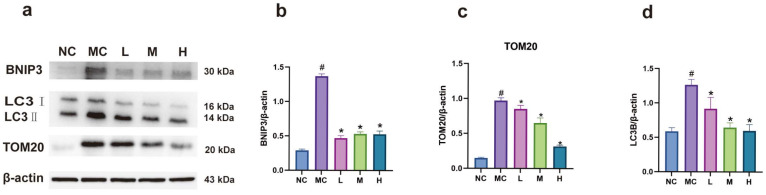
(**a**) Western blot band image; (**b**–**d**) bar charts depicting statistical data on the expression of autophagy-related proteins: NC: normal group, MC: model group (CoCl_2_: 300 μM), L: low-dose emodin group (CoCl_2_: 300 μM, Emo: 5 μM), M: medium-dose emodin group (CoCl_2_: 300 μM, Emo: 10 μM), H: high-dose emodin group (CoCl_2_: 300 μM, Emo: 20 μM). Compared to the normal group, # *p* < 0.05 for the model group; compared to the model group, * *p* < 0.05 for the emodin groups.

Western blot results indicated that, compared to the normal group, the expression of BNIP3 and LC3B proteins was significantly increased in the model group (*p* < 0.05). In contrast, the expression of BNIP3 and LC3B proteins was significantly decreased in each emodin dosage group compared to the model group (*p* < 0.05). There was no significant difference in BNIP3 expression among the emodin dosage groups (*p* > 0.05). Compared to the low-dose group, the expression of LC3B and TOM20 proteins significantly decreased in the medium-dose and high-dose emodin groups (*p* < 0.05), with no significant difference between the medium- and high-dose groups (*p* > 0.05). These findings suggest that emodin inhibits mitochondrial autophagy via the HIF-1/BNIP3 pathway (see Figure 8).

## 4. Discussion

RA is a chronic inflammatory autoimmune disease characterized by involvement of multiple joints and a high rate of disability [17]. Its typical pathological features include progressive aggravation of joint synovitis and irreversible structural damage to joints; however, its exact etiology and pathogenesis remain incompletely understood.

Pyroptosis is a crucial form of programmed cell death marked by progressive cell swelling that leads to plasma membrane rupture, accompanied by the release of intracellular pro-inflammatory factors and triggering a cascade of inflammatory responses [18]. The molecular mechanism primarily involves the recruitment and activation of Caspase-1 by the NLRP3 inflammasome via the ASC adaptor protein, which cleaves Gasdermin D (GSDMD) to form plasma membrane pores and simultaneously matures inflammatory cytokines such as IL-1β and IL-18 [19]. Notably, NLRP3 activation can be triggered by various pathological stimuli, including ATP fluctuations, ion gradient imbalances [20,21], pathogen-associated RNA, and bacterial and fungal toxins and components [22]. Additionally, abnormal accumulation of reactive oxygen species related to mitochondrial dysfunction and lysosomal stability disruption have been confirmed as significant triggers for inflammasome activation [23].

Mitophagy, as a key mitochondrial quality control mechanism, maintains cellular homeostasis by selectively removing damaged mitochondria [24]. Studies have shown that the continuous release of IL-1β and IL-18 is closely related to the balance between the HIF-1α/NLRP3 and HIF-1α/BNIP3 pathways. It is noteworthy that the combined effect of mitophagy and pyroptosis leads to inflammatory changes in synovial fibroblasts (FLS), and through the continuous secretion of inflammatory factors such as IL-1β and IL-18 ultimately promotes the progression of synovitis [6].

Previous clinical and basic experimental results from our team have shown that the external application of Compound Rhubarb Powder, with rhubarb as the principal herb, can significantly reduce joint redness, swelling, heat, and pain in RA patients. Its active ingredient, emodin, significantly inhibits arthritis inflammation in CIA rats, improving pathological changes such as joint bone marrow edema and bone erosion. One mechanism involves the inhibition of inflammatory pathways such as MAPK and NF-κB [12,13,14,15]. Notably, this study utilized a CoCl_2_-induced chemical hypoxia model to simulate the RA synovial microenvironment, and the inhibition rate data reflect the characteristics of the in vitro model. Clinical studies have widely reported reduced oxygen partial pressure and abnormal cell metabolism in the synovial tissues of RA patients [3,4]. However, specific changes in cell viability need comprehensive analysis in conjunction with individual differences and disease stages.

Hypoxia-inducible factors (HIFs), particularly HIF-1α, play a crucial role as mediators in the hypoxic response, causing pyroptosis through the HIF-1α/NLRP3 pathway and exacerbating the inflammatory response [11]. Electron microscopy observations indicate that mitochondrial dysfunction caused by this pathway is a major reason for inflammation [25]. Some studies have found that emodin induces mitophagy through the PINK1/Parkin pathway [26]. However, this study found that emodin inhibits mitophagy through the HIF-1α/BNIP3 pathway, regulating the balance between pyroptosis and mitophagy. This change may be related to hypoxia modeling, emodin dosage, and the duration of emodin action, and the specific conditions required for autophagy need further study.

Results of this study showed the following: 1. MH7A cell viability significantly decreased after CoCl_2_ induction, characterized by cell swelling with numerous bubble-like protrusions, mitochondrial swelling, endoplasmic reticulum expansion, and other pathological changes. Secretion levels of HIF-1α, IL-1β, and IL-18 significantly increased, as did the expression of related proteins such as HIF-1α, NLRP3, GSDMD, BNIP3, and LC3B. Additionally, the immunofluorescence intensity of NLRP3 was significantly enhanced. This suggests that HIF-1α, as a key regulator of synovial hypoxia, simultaneously triggers mitophagy and NLRP3 inflammasome activation, playing a crucial role in the occurrence and development of RA synovitis. 2. After emodin intervention, the levels of HIF-1α, IL-1β, and IL-18 in MH7A cells significantly decreased, as did the expression of related proteins such as HIF-1α, NLRP3, GSDMD, BNIP3, and LC3B. The immunofluorescence intensity of NLRP3 also significantly weakened. This indicates that emodin can inhibit mitophagy, regulate the balance between the HIF-1α/BNIP3 and HIF-1α/NLRP3 pathways, and inhibit RA synovitis by suppressing NLRP3 inflammasome activation.

These study results suggest that HIF-1α, as a key regulator of synovial hypoxia, triggers NLRP3 inflammasome activation, inducing RA synovitis. Emodin can dynamically regulate the balance between mitophagy and pyroptosis through the HIF-1α molecular switch, thereby inhibiting RA synovitis. However, a limitation of this study is the absence of a control group with a specific HIF-1α inhibitor. Future research will incorporate gene silencing technology to further investigate the regulatory mechanism of the HIF-1α/NLRP3 pathway.

This study reveals for the first time that emodin exerts anti-RA effects through dual regulation of HIF-1α/BNIP3-mediated mitophagy and HIF-1α/NLRP3-mediated pyroptosis. However, several issues require attention: further analysis is needed to understand the synergistic and antagonistic effects of emodin with other components in traditional Chinese medicine formulations; validation through animal models and clinical translation studies is urgently required; the spatiotemporal regulation mechanisms underlying the dynamic balance of HIF-1α still need in-depth exploration; and MH7A cells, as immortalized cells, cannot fully replicate the complex environment of the human body. Future research will integrate in vitro and in vivo models with single-cell sequencing technology to systematically elucidate the multi-target regulatory network of natural compounds, providing theoretical evidence for developing modernized preparations of traditional Chinese medicine based on clear “component-target-pathway” relationships.

## Data Availability

The data have already been included in the article or submitted as an attachment.
